# Improving Pulmonary Infection Diagnosis with Metagenomic Next Generation Sequencing

**DOI:** 10.3389/fcimb.2020.567615

**Published:** 2021-01-26

**Authors:** Yi-Yi Qian, Hong-Yu Wang, Yang Zhou, Hao-Cheng Zhang, Yi-Min Zhu, Xian Zhou, Yue Ying, Peng Cui, Hong-Long Wu, Wen-Hong Zhang, Jia-Lin Jin, Jing-Wen Ai

**Affiliations:** ^1^ Department of Infectious Diseases, Huashan Hospital, Fudan University, Shanghai, China; ^2^ BGI PathoGenesis Pharmaceutical Technology Co., Ltd, BGI-Shenzhen, Shenzhen, China; ^3^ BGI Wuhan Biotechnology, BGI-Shenzhen, Wuhan, China; ^4^ State Key Laboratory of Genetic Engineering, School of Life Science, Fudan University, Shanghai, China; ^5^ National Clinical Research Center for Aging and Medicine, Huashan Hospital, Fudan University, Shanghai, China; ^6^ Key Laboratory of Medical Molecular Virology (MOE/MOH) and Institutes of Biomedical Sciences, Shanghai Medical College, Fudan University, Shanghai, China

**Keywords:** pulmonary infection, metagenomic, next generation sequencing, diagnosis, respiration tract infection

## Abstract

Pulmonary infections are among the most common and important infectious diseases due to their high morbidity and mortality, especially in older and immunocompromised individuals. However, due to the limitations in sensitivity and the long turn-around time (TAT) of conventional diagnostic methods, pathogen detection and identification methods for pulmonary infection with greater diagnostic efficiency are urgently needed. In recent years, unbiased metagenomic next generation sequencing (mNGS) has been widely used to detect different types of infectious pathogens, and is especially useful for the detection of rare and newly emergent pathogens, showing better diagnostic performance than traditional methods. There has been limited research exploring the application of mNGS for the diagnosis of pulmonary infections. In this study we evaluated the diagnostic efficiency and clinical impact of mNGS on pulmonary infections. A total of 100 respiratory samples were collected from patients diagnosed with pulmonary infection in Shanghai, China. Conventional methods, including culture and standard polymerase chain reaction (PCR) panel analysis for respiratory tract viruses, and mNGS were used for the pathogen detection in respiratory samples. The difference in the diagnostic yield between conventional methods and mNGS demonstrated that mNGS had higher sensitivity than traditional culture for the detection of pathogenic bacteria and fungi (95% vs 54%; p<0.001). Although mNGS had lower sensitivity than PCR for diagnosing viral infections, it identified 14 viral species that were not detected using conventional methods, including multiple subtypes of human herpesvirus. mNGS detected viruses with a genome coverage >95% and a sequencing depth >100× and provided reliable phylogenetic and epidemiological information. mNGS offered extra benefits, including a shorter TAT. As a complementary approach to conventional methods, mNGS could help improving the identification of respiratory infection agents. We recommend the timely use of mNGS when infection of mixed or rare pathogens is suspected, especially in immunocompromised individuals and or individuals with severe conditions that require urgent treatment.

## Introduction

Pulmonary infection is a type of respiratory tract infection (RTI) that may lead to various complications and is associated with a high mortality rate worldwide (Magill et al., 2014). Pulmonary infections are cause by a wide variety of pathogens, including bacteria, fungi, viruses, and parasites, alone or in combination. Therefore, accurate and timely diagnosis of the cause of the infection is crucial to enable the appropriate treatment of pulmonary infection and improved outcomes, especially among patients who need combined treatment for co-infections (Hardak et al., 2016).

In current clinical practice, conventional methods for diagnosing the cause of infection include microbial culture, serology, antigen/antibody assays, and polymerase chain reaction (PCR)-based nucleic acid detection (Loeffelholz and Chonmaitree, 2010; Labelle et al., 2010). Nevertheless, the diagnostic efficiency of these methods is hindered by the high diversity of RTI pathogens, as well as the presence of commensal microbiota and pathobionts in the respiratory tract (Huffnagle et al., 2017). For example, microbial culture has a long turn-around time (TAT) and is unable to detect viruses and parasites, while antigen/antibody assays may have limited sensitivity (Loeffelholz and Chonmaitree, 2010). Although conventional PCR-based nucleic acid detection has high sensitivity and specificity, it detects a limited range of microorganisms which may not include the pathogen responsible for the infection. Therefore, pathogen detection and identification methods for pulmonary infection with higher diagnostic efficiency are urgently needed to overcome the limitations in sensitivity, specificity, TAT, and diagnostic spectrum.

Unbiased metagenomic next generation sequencing (mNGS) has been used for the detection of infectious pathogens, especially for detecting rare or newly emergent pathogens (Lu et al., 2020), and exhibits better diagnostic performance than traditional methods (Miao et al., 2018; Zhang et al., 2019). mNGS analysis is capable of simultaneously detecting thousands of pathogens using a diverse range of specimen types (Ai et al., 2018; Wilson et al., 2019; Zhang et al., 2019; Xing et al., 2020; Zhang H. C. et al., 2020), and has the potential to substantially increase the diagnostic efficiency. Miao *et al.* reported that the mNGS had higher sensitivity and specificity than microbial culture, especially for the detection of *Mycobacterium tuberculosi*s, viruses, anaerobic bacteria, and fungi (Miao et al., 2018). Another study by Zhou et al. demonstrated that the performance of mNGS was less affected by prior antibiotic exposure than culture (Zhou et al., 2019). Although mNGS has been applied in the diagnosis of RTI using a range of specimen types (Li et al., 2018; Zhou et al., 2019), there have been few comprehensive studies of the performance and value of mNGS for diagnosing pulmonary infections.

In this study, we compared the diagnostic yield between mNGS and conventional methods, and evaluated the clinical impact of mNGS in the diagnosis of pulmonary infections.

## Methods

### Study Design and Participants

**Figure 1:**
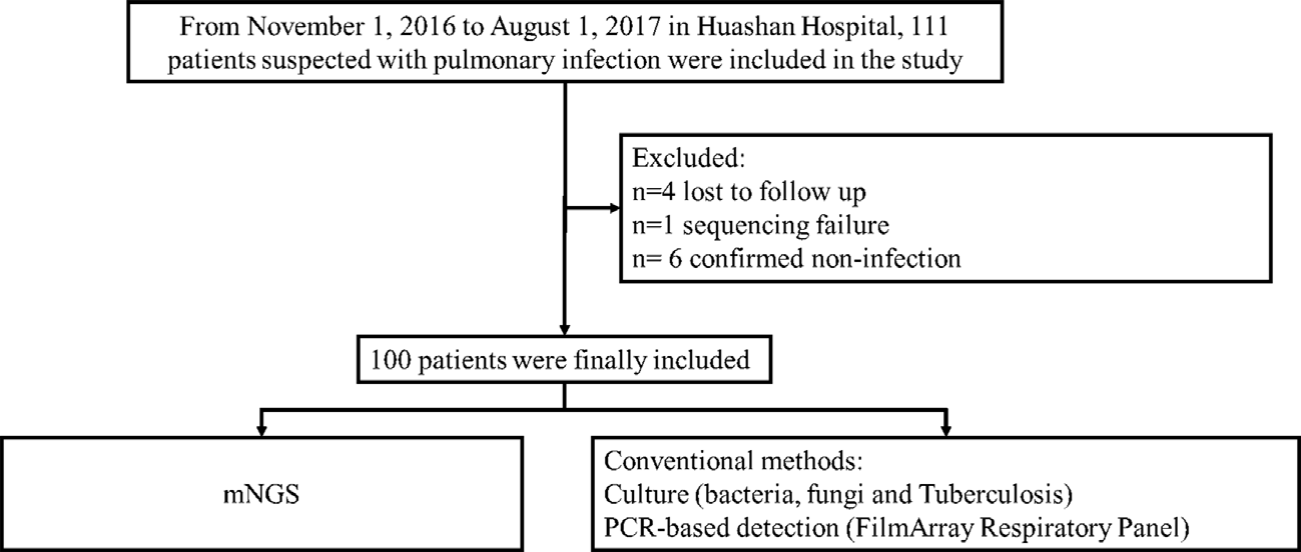
Overview of patient enrollment workflow.

This single-center prospective observational study was conducted in Huashan Hospital, Shanghai, China from November 1, 2016 to August 1, 2017. Patients who met the following criteria were enrolled: (1) exhibited typical clinical signs of pulmonary infection such as fever, cough, expectoration, and respiratory failure; and (2) the diagnosis of pulmonary infection was supported by radiological evidence, including the result of chest X-ray or computed tomography scan. Those in whom the diagnosis of an infection was ruled out and those who were lost to follow-up were excluded from the cohort. The recruitment process is shown in **Figure 1**. Respiratory tract samples, including nasopharyngeal swabs (NPS), sputum, and bronchoalveolar lavage fluid (BALF), were collected from patients within 24 hours (NPS and sputum) or 48 hours (BALF) of admission or disease onset.

The samples were sent for culture and smear tests (culture medium: bacteria: blood agar plates/chocolate agar plates/MacConkey agar; fungi: Sabouraud Dextrose Agar; Mycobacterium: Roche medium. Condition: 35°C, with 5% CO_2_). Other conventional pathogen detection tests, such as the FilmArray Respiratory Panel (FA-RP), were conducted as required. Duplicate specimens were later submitted for mNGS analysis.

Informed consent was obtained for each patient prior to enrolment. The study was approved by the ethics review committee of Huashan Hospital (No. Ky2017-338). Patients’ medical records were reviewed to collect baseline information, including age, sex, presence of immunosuppressive conditions, onset site, whole blood cell count, C-reactive protein and procalcitonin level, smear test results, culture, and other microbiological results, and the patients’ treatment regime.

### Metagenomic Next Generation Sequencing and Data Analysis

NPS, sputum, and BALF samples from patients were collected according to standard operating procedures. Each tip of the NPS was immersed in 3 mL preservation medium (UTM-RT transport medium, COPAN Diagnostics Inc, CA, USA). DNA extraction was conducted for each sample, while RNA extraction and reverse transcription were applied according to the patient’s manifestations at the discretion of the physician’s clinical decisions, particularly if a viral infection was suspected.

For DNA extraction, 1.5 mL microcentrifuge tubes each containing a 0.6 mL sample or immersing preservation medium and 1g 0.5mm glass bead were attached to a horizontal platform on a vortex mixer and agitated vigorously at 2800-3200 rpm for 30 min. Then a 0.3mL sample was separated into a new microcentrifuge tube, and the total DNA was extracted using the TIANamp Micro DNA Kit (DP316, TIANGEN BIOTECH) according to the manufacturer’s recommendation. For RNA extraction, total RNA were extracted from the 0.3 mL sample or immersing preservation medium by QIAamp ViralRNA Mini KIT(52904#, QIAGEN). The complementary DNA (cDNA) was generated from RNA templates by reverse transcription using SuperScript™ II Reverse Transcription Kit (18064-014 Invitrogen), followed by the synthesis of the second strand. The total DNA or cDNA was subjected to library construction through DNA-fragmentation (150bp), end-repair, adapter-ligation, and unbiased PCR amplification. Agilent 2100 was used for quality control of the DNA libraries (200-300bp). Quality qualified libraries were sequenced by BGISEQ-50 platform (Jeon et al., 2014).

After removing low-quality reads (< 35 bp) and computational subtracting human host sequences mapped to the human reference genome (hg19) from the sequencing data by Burrows-Wheeler Alignment (0.7.10-r789) (Li and Durbin, 2009), high-quality sequences were generated. Following the removal of low-complexity reads according to prinseq (version 0.20.4), the remaining sequences were phylogenetically classified by aligning to PMDB (PMseq metagenomic Database, version 3.0, BGI-locally established database) consisting of 2,700 whole genome sequences of viral taxa, 1,494 bacterial genomes or scaffolds, 73 fungi related to human infection, and 47 parasites associated with human diseases, which were downloaded from NCBI (ftp://ftp.ncbi.nlm.nih.gov/genomes/).

Using JX625134.1 as the reference genome, two adenovirus B1 genomes were assembled with SPAdes-3.12.0. Thirty-six human adenovirus B genomes of high identity (percent identity ≥ 96.9%) with the assembled adenovirus B1 genomes, and thirteen human adenovirus reference genomes of different serotypes from NCBI Reference Sequence database were downloaded for phylogenetic analysis as outgroup. Single copy genes were identified following genome annotation (by Prokka v1.12), gene alignment (by blastall v2.2.25) and clustering. Human adenovirus genomes were aligned using the single copy genes as conserved regions by MUSCLE v3.7 (Edgar, 2004). Phylogenetic analyses of the conserved regions were conducted by PhyML software v3.0 (Guindon et al., 2010) using Maximum likelihood method, with the HKY85 substitution model and gamma distribution rates model as the chosen parameters. The reliability of each nodes was estimated by aLRT method with non-parametric SH branch support mode.

### Criteria for a Positive mNGS Result

The sequencing results of each sample were categorized into 2 tables, each presenting bacteria/fungi and virus, respectively. The specifically mapped read number (SMRN) of each microbial taxonomy was normalized to SMRN per 20 million (M) of total sequencing reads (SDSMRN, standardized SMRN).

$$\text{SDSMRN}=\frac{\text{SMN}}{\text{Total reads}}\times 20\;million$$

A virus was considered positively detected if: 1) it was among the top 3 viruses with highest SDSMRN; and 2) it had a SDSMRN > 5. A bacterial/fungal species was considered positively detected if: 1) it belonged to the top 10 genera with the highest SDSMRN; 2) it ranked first within its genus; 3) it had a SDSMRN>1; and 4) it was a commonly reported pulmonary infectious pathogen.

However, there were several exceptions for certain pathogens. For the detection of *Mycobacterium* spp., *Nocardia* spp., Brucella spp., etc., because of the difficulty of DNA extraction and low possibility for contamination, the pathogen was considered detected if:1) its genus was among the top 20 with highest SDSMRN; 2) it ranked first within its genus; and 3) it had a SDSMRN>1. For the detection of pathogens within *Enterobacteriaceae* family, only the species with highest SDSMRN was considered as a positive detection.

### Conventional Microbiological Analysis

Samples parallel underwent conventional microbiological methods and mNGS. All of the collected samples were sent to the clinical laboratory, Huashan Hospital for culture and smear tests of bacteria, fungi, and mycobacteria. Blood agar plates, chocolate agar plates and MacConkey agar were used for culture of bacteria, with the temperature of 35°C and 5% CO_2_. Roche medium were for mycobacterium. Sabouraud Dextrose Agar were used for fungi, with the temperature of 37°C and 25°C. A FilmArray Respiratory Panel (FA-RP, Biofire, Salt Lake City, UT, USA) was employed for nucleic acid detection if the suspected pathogens were within the detection targets, which consisted of adenovirus, coronavirus (strains HKU1, NL63, 229E, OC43), human metapneumovirus, rhinovirus/enterovirus, influenza (strains A, A/H1, A/H3, A/H1-2009, B), parainfluenza virus (strains 1,2, 3, 4), and respiratory syncytial virus (RSV) as well as the bacterial respiratory pathogens *Mycoplasma*, *B pertussis*, and *Chlamydophilia*.

### Statistical Analysis

The chi-square test was applied to assess the pathogen-specific diagnostic performance of each method, reported as sensitivity, specificity, positive predictive value and negative predictive value with their 95% confidence intervals (95% CI). Statistical analysis and figure drawings were performed using the SPSS statistical package 20.0 software and GraphPad Prism 5 software. P values< 0.05 were considered statistically significant.

## Results

### General Characteristics of the Enrolled Cohort

A total of 111 patients with suspected pulmonary infections consented to sample collection and were clinically screened (**Figure 1**). Of these patients, 4 were lost to follow-up, 1 did not receive any mNGS results, and 6 were confirmed to have non-infectious conditions, resulting in the final enrollment of 100 patients. Their baseline characteristics are shown in **Table 1**. Respiratory samples from these patients were tested using next generation sequencing as well as traditional methods.

**Table 1:** Patients’ baseline characteristics.

	Total patients group (n=100)
Male(%)	65(67.7)
Age(mean ± SD)	54.1 ± 18.25
Clinical feature	
White Blood Cells (×109/L)	6.8 ± 3.8
Neutrophil (%)	73.0 ± 17.0
Lymphocyte (%)	17.3 ± 11.7
CRP^1^ (mg/L)	56.1 ± 54.9
PCT^2^ (ng/mL)	0.19 (0.08, 0.46)^3^
Immunity status (%)	
Compromised	26 (26.8)
Normal	67 (69.1)
Unclear	4 (4.1)
Onset location (%)	
Community	81 (83.5)
Hospital	14 (14.4)
Unclear	2 (2.1)

^1.^CRP, C-Reactive Protein.

^2.^PCT, Procalcitonin.

^3.^Median (25% quartile, 75% quartile).

### Diagnosis Performance of mNGS in Virus Detection

**Figure 2:**
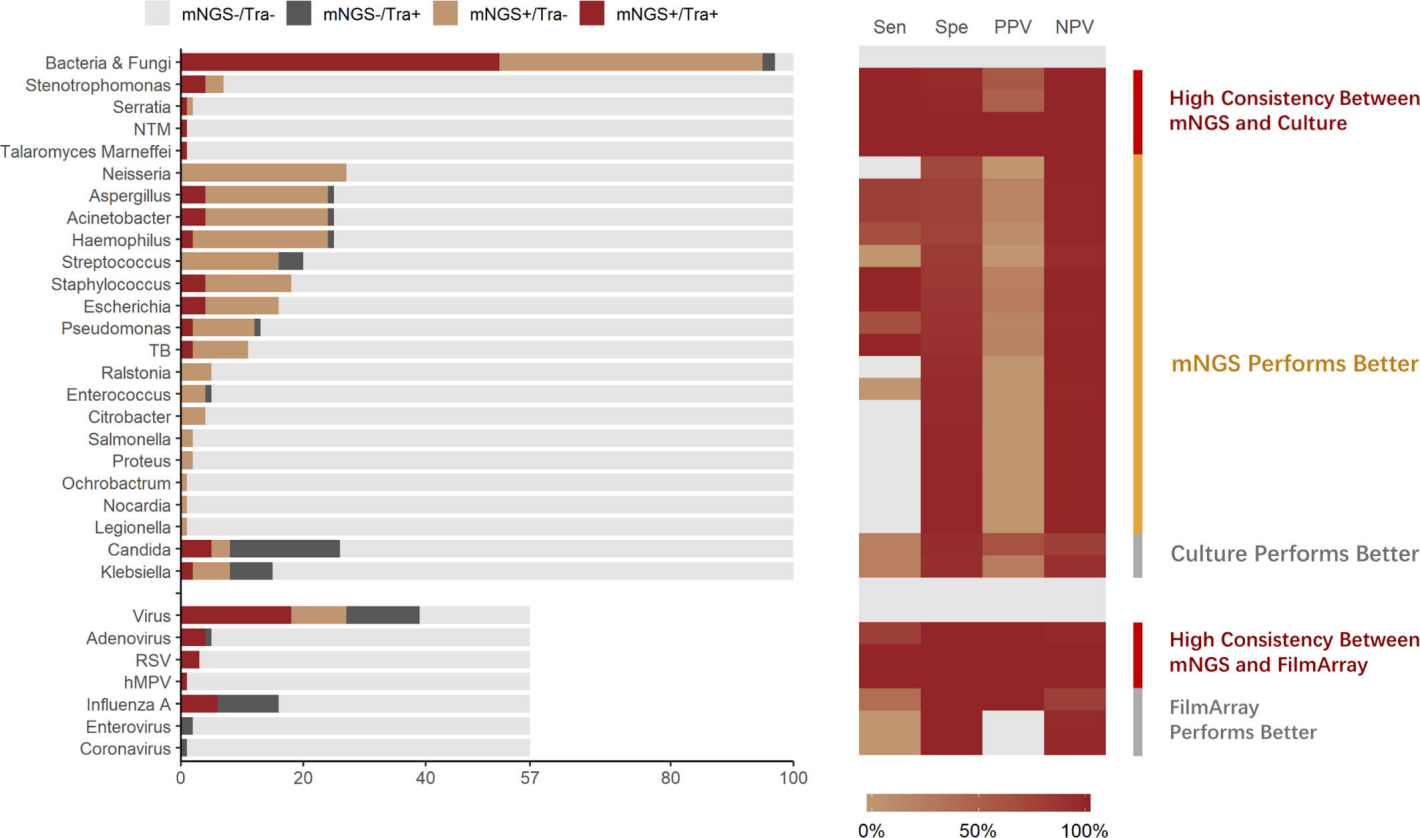
Distribution and diagnostic performance of identified pathogens in mNGS and traditional pathogen detection. Tra, Traditional Pathogen Detection Methods, culture for Bacteria and Fungi, FilmArray for Virus; NTM, Nontuberculous Mycobacteria. TB, Tuberculosis. RSV, Respiratory Syncytial Virus. hMPV, human Metapneumovirus; Sen, Sensitivity; Spe, Specificity; PPV, Positive Prediction Value; NPV, Negative Prediction Value.

In total, 57 samples were tested by both mNGS and FA-RP. The FA-RP detected 28 respiratory viruses in 26 samples (26/57, 45.6%), while mNGS detected 26 viruses in 24 samples (24/57, 42.1%). The FA-RP-detected viruses included adenovirus (ADV, n=5), human metapneumovirus (hMPV, n=1), influenza A virus (n=16), human rhinovirus (HRV, n=2), respiratory syncytial virus (RSV, n=3) and coronavirus HKU1 (n=1). Of these viruses, 50% (14/28) were also detected by mNGS according to the criteria of positive detection (**Figure 2**). There was a high level of agreement between FA-RP and mNGS in the detection of ADV, RSV, and hMPV, with a sensitivity of 80.0%, (95% CI: 29.9–98.9%), 100%, (95% CI: 31.0–100%), 100%, (95% CI: 5.5–100%), respectively (**Figure 2**). However, a total of 14 viruses that were detected by FA-RP were not detected using mNGS (**Table 2**). Among these false-negative cases, 28.6% (4/14) were detected by mNGS without meeting the criteria of positive detection, while the remaining 71.4% (10/14) were not detected at all (**Table 2**). mNGS had lower sensitivity in the detection of influenza A and rhinovirus, with a sensitivity of 37.5% (95% CI: 16.3–64.1%) and 0% (95% CI: 0.0–80.2%), respectively (**Figure 2**). A case of coronavirus HKU1 was not detected using mNGS because the sample only went through the DNA extraction procedure, which did not include an RNA reverse transcription process to identify RNA viruses. In addition, the mNGS detected another 14 viruses, including cytomegalovirus (CMV, n=5), herpes simplex virus (HSV, n=3), Epstein-Barr virus (EBV, n=6), which are beyond the detection targets of FA-RP. Among these extra viruses’ detections, 3 CMV (3/5) detections were considered to cause the infection, while the others were not considered clinically relevant because of their uncertainty of pulmonary pathogenicity. In conclusion, although mNGS had lower sensitivity than FA-RP PCR for the diagnosis of common respiratory viruses, it has potential of identifying extra viruses that were not detected by conventional methods.

**Table 2:** Pathogens detected by conventional methods but missed by mNGS.

Possible Explanation					
Microbe	Number	Not detected	Detected without meeting the criteria		No Reverse transcription
*Acinetobacter*	1	1			
*Haemophilus*		1		1		
*Pseudomonas*		1		1		
*Streptococcus*		4		4		
*Aspergillus*		1	1			
*Candida*		18	5	13		
*Klebsiella*		7	1	6		
*Enterococcus*		1		1		
Rhinovirus		2	1	1		
Influenza A		10	8	2		
Coronavirus		1			1	
Adenovirus		1		1		
Total		48	17	30	1	

### Diagnosis Performance of mNGS in Bacteria and Fungi Detections

All samples underwent both mNGS and culture. Culture identified 70 pathogenic bacterium or fungus in 54 samples (54.0%), while mNGS identified a total of 225 pathogenic bacterium or fungus in 95 samples (95.0%), of which the positive rate is significantly higher (*p*<0.001). The culture-detected pathogens included *Streptococcus* spp. (n=4), *Stenotrophomonas* spp. (n=4), *Staphylococcus* spp. (n=4), *Serratia* spp. (n=1), *Pseudomonas* spp. (n=3), *Talaromyces marneffei* (n=1), *Klebsiella* spp. (n=9), *Haemophilus* spp. (n=3), *Escherichia* spp. (n=4), *Enterococcus* spp. (n=1), *Aspergillus* spp. (n=5), *Candida* spp. (n=23), *Acinetobacter* spp. (n=5), non-tuberculous *Mycobacteria* (n=1), and *Mycobacterium tuberculosis* (n=2). Of these microbes, 51.4% (36/70) were identified using mNGS. High consistency between these 2 methods was observed in the detection of pathogens such as *Stenotrophomonas* spp, (sensitivity: 100%, 95% CI: 39.6–100%) (**Figure 2**). In the detection of pathogens such as *M. tuberculosis* and *Staphylococcus* spp., mNGS performed better than culture. mNGS identified all the M. tuberculosis (2/2, 100%) and *Staphylococcus* spp. (4/4, 100%) detected by culture, with additional detection of 9 *M. tuberculosis* and 14 *Staphylococcus* spp. strains in culture-negative samples (**Figure 2**).

Nevertheless, a total of 34 bacteria/fungi tested positive by culture failed to be reported by mNGS (**Table 2**). Among these false-negative cases, 76.5% (26/34) were detected by mNGS without meeting the criteria of positive detection, while the remaining 23.5% (8/34) were not detected at all (**Table 2**). Particularly, mNGS had inferior detection performance of *Candida* spp. and *Klebsiella* spp. Only 21.7% (5/23) of the *Candida* spp. and 22.2% (2/9) of the *Klebsiella* spp. strains were reported by mNGS in culture-positive samples (**Figure 2**). In conclusion, although mNGS had a better positive rate than culture in bacteria and fungi detections, its performance highly varied across different pathogens.

### Extra Detection of Pathogens by mNGS

To explore the “false-positive” results of mNGS against conventional methods, patients’ clinical data were analyzed thoroughly by two experienced physicians. In total, mNGS identified 183 culture-negative pathogens, including *Neisseria* spp. (n=27), *Haemophilus* spp. (n=22), *Aspergillus* spp. (n=20), *Acinetobacter* spp.(n=20), *Streptococcus* spp. (n=16), *Staphylococcus* spp. (n=14), *Escherichia* spp. (n=12), *Pseudomonas* (n=10), *Mycobacterium tuberculosis* (n=9), *Klebsiella* (n=6), *Ralstonia* (n=5), *Enterococcus* (n=4), *Citrobacter* (n=4), *Stenotrophomonas* (n=3), *Candida* (n=3), *Salmonella* (n=2), *Proteus* (n=2), *Serratia* (n=1), *Ochrobactrum* (n=1), *Nocardia* (n=1), *Legionella* (n=1). Among them, although most were considered as respiratory tract commensal microbiome or contamination, mNGS identified 16 extra bacterial/fungal pathogens with high relevance to clinical manifestations, including 3 culturable bacteria (e.g. *Enterococcus faecium*, *Escherichia coli* and *Klebsiella pneumoniae*), 6 fastidious bacteria (e.g. *Mycobacterium tuberculosis*, *Nocardia* spp. etc.), and 7 bacteria (e.g. *Mycoplasma pneumoniae*, *Chlamydia psittaci* etc.) which are unculturable under standard conditions. On the other hand, among 14 mNGS-positive/FA-RP-negative viruses, 3 CMV detections were considered clinically relevant. This result indicates that in diagnosis of pulmonary infection, mNGS may report a massive amount of clinically irrelevant pathogens. However, it could improve the diagnosis yield that might actually benefit the clinical decision.

### Application of the mNGS Data in Genomic Analysis

In addition to pathogen detection, mNGS data could also provide genetic information for epidemiology analysis. As viral genomes are relatively small in size, good genome coverage and sequencing depth were achieved in the detection of certain viral strains for further analysis. In this study, a total of 45 viruses were reported positive by mNGS, of which 13.3% (6/45) had qualified genome coverage (over 90%) and sequencing depth (over 30 ×) for whole genome assembling and genomic analysis. These 6 viral strains consisted of adenovirus B1 (n=4), influenza virus A (n=1) and HPV-4 (n=4). As representative, adenovirus B1 detected in 2 samples with high genome coverage (over 95%) and sequencing depth (over 180 ×) were used for genome assembling and analysis. Both adenovirus B1 genomes were assembled from samples collected in 2017.

**Figure 3:**
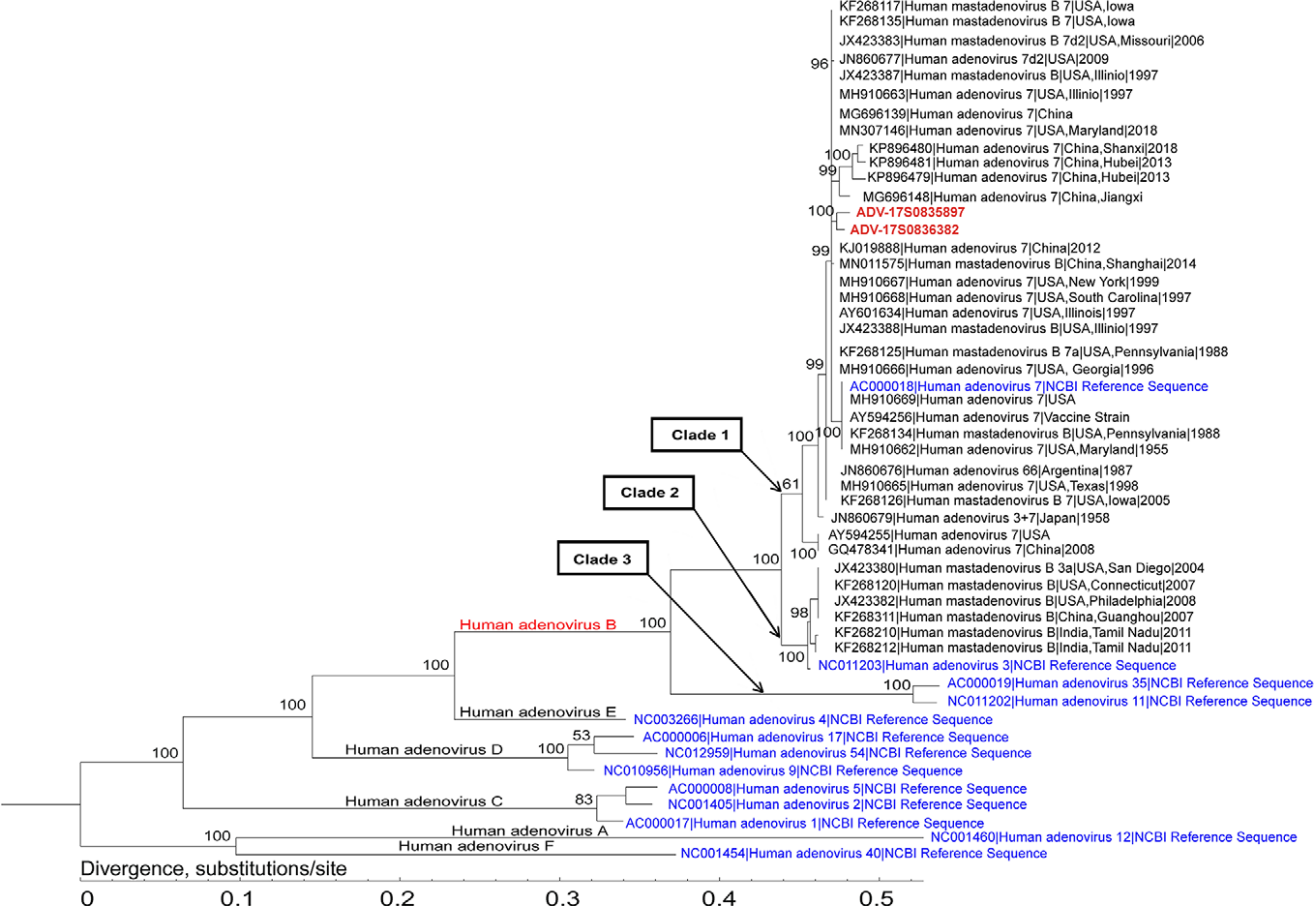
Phylogenetic analysis of the representative adenovirus B1 genomes. This analysis involved 2 newly assembled adenovirus B1 genomes, 36 published human adenovirus B genomes, and 13 human adenoviruses from NCBI Reference Sequence database. The two adenovirus B1 genomes (ADV-17S0835897 and ADV-17S0836382) were located in the same branch, and had high genetic similarity with strains identified in China.

Phylogenetic analysis of the assembled adenovirus B1, its closely related genomes, as well as reference genomes, revealed that six species of Mastadenovirus (Human mastadenovirus A-F) formed six branches (**Figure 3**). The Human adenovirus B branch could be classified into three clades: the two strains in this study (ADV-17S0835897 and ADV-17S0836382) and 30 Human adenovirus 7 strains, as well as the Human adenovirus 7 reference genome formed Clade 1; Clade 2 was formed by six Human adenovirus 3 strains and the Human adenovirus 3 reference genome; and Human adenovirus 35 and Human adenovirus 11 references genomes formed Clade 3. The ADV-17S0835897 and ADV-17S0836382 had high genetically similarity with a strain (MG696148) described as a possible cause of a cluster severe acute respiratory infections in Jiangxi province, China, and three strains (KP896479, KP896480 and KP896481) related to an outbreak of febrile respiratory illness in Hubei, China. Since the patients from whom ADV-17S0835897 and ADV-17S0836382 were detected had no contact history with each other, the high genetic similarity of these two stains indicated that there might be an epidemic adenovirus B strain in 2017.

## Discussion

Accurate and fast pathogen detection is essential for the management of RTIs. Although previous studies have reported the use of mNGS for the identification of respiratory pathogens, few studies have comprehensively evaluated the overall diagnostic performance of mNGS in RTI. This cross-sectional study evaluated the diagnostic yield and extra diagnostic value of mNGS in RTI.

Although mNGS is a good supplement to the current pathogen detection methods, its diagnostic performance has limitations. mNGS had lower sensitivity than PCR in the detection of certain viruses, such as influenza A and rhinovirus. When FA-PR was used as the referent, the sensitivity and specificity of mNGS were 50% and 100%, and mNGS failed to identify 14 viruses. Other studies have had similar findings (Prachayangprecha et al., 2014; Thorburn et al., 2015). Thorburn *et al.* reported that a mNGS had a sensitivity and specificity of 78% and 80%, respectively, with RT-PCR as referent, and attributed the limited sensitivity of mNGS to the low abundance of the 11 undetected respiratory viruses, which is consistent with the findings of Prachayangprecha et al. Those similar conclusions from different studies suggested that mNGS might not be as sensitive as PCR detecting respiratory viruses. As the sensitivity of mNGS is significantly impacted by the sequencing depth, theoretically, increasing the total number of sequencing reads per sample could improve its sensitivity, and cost as well. In contrast to the above findings, mNGS detected an additional 14 viruses that were beyond the detection targets of FA-RP. Among them, 3 CMV were confirmed to cause infection in immuno-compromised patients, which indicates mNGS’s potential in the detection of rare and unexpected viruses, especially under special circumstances. In conclusion, although mNGS with current depth and procedure cannot replace PCR for the diagnosis of common viral RTIs, its unbiased detection enables the identification of viral pathogens that are undetectable using conventional PCR panels.

In the detection of pathogenic bacteria and fungi, although the performance of mNGS varied across different pathogens, it detected significantly more potential pathogens than culture in this study. In our practice, a total of 183 culture-negative pathogens were identified by mNGS. Among them, 16 bacterial/fungal pathogens were considered highly clinically relevant, including fastidious pathogens such as *M. tuberculosis* and *Nocardia* spp., which may require a long incubation time, as well as some unculturable pathogens under standard conditions such as *Mycoplasma pneumoniae*. mNGS, with a relatively short TAT and untargeted nature, was capable of detecting those pathogens quickly. Considering these results, mNGS may serve as an important supplement to current conventional culture, and improve the pathogen detection and disease management of patients with complex infectious conditions (Parize et al., 2017; Pan et al., 2019; Wang et al., 2020). Similar conclusions have been made from previous studies. When used as a supplementary method to culture, mNGS increases the diagnostic yield, in focal and central nervous system infections (Zhang et al., 2019; Zhang Y. et al., 2020).

Nevertheless, mNGS was unable to detect certain culturable pathogens. In this study, mNGS missed 34 bacteria/fungi tested positive by culture, and performed poorly in the detection of pathogens such as *Klebsiella pneumoniae*. It is possibly due to the hindrance of the commensal microbiome in the respiratory tract (Wypych et al., 2019). Another limitation of mNGS is that it is unable to discriminate the pathogenicity status of the pathobionts detected. In this study, mNGS identified 225 pathogenic bacterium or fungus and 26 viruses in 100 samples, most of which belong to the respiratory tract commensal microbiome or contamination and were not clinically relevant. Such large amounts of information could be confusing and even misleading to physicians while making clinical decisions. Furthermore, a standard criterion for the interpretation of mNGS results, such as the definition of “positive or negative”, is still lacking, which may also affect the clinical use of mNGS. In conclusion, although mNGS is not suitable for use as the sole diagnostic method for RTIs, it could improve diagnostic efficiency and serve as a supplementary method to culture. However, the interpretation of mNGS results can be rather confusing presently.

mNGS is capable of providing genetic and genomic information with significance in epidemiologic analysis. Since mNGS can provide information on the genetic sequence of the detected pathogens, its application for identifying newly emergent and rare pathogens, has been widely acknowledged (Lu et al., 2020; Zhu et al., 2020). With its genome assembly and genomic analysis procedure, the genetic information provided by mNGS could be further applied in evolutionary and epidemiologic studies. Our results showed that at a total sequencing depth of 20M reads/sample, over 10% of the detected respiratory viruses had adequate genome coverage and depth for further genomic and epidemiologic analysis. Since fungi and bacteria have larger genomes, our study did not achieve whole genome assembly of fungal and bacterial pathogens. However, the assembly of marker genes or partial genomes with mNGS data for genomic analysis has been reported (Zhu et al., 2018).

This study had limitations. First, the sample size was limited, which might have affected the accuracy of the evaluation of the performance of mNGS. Second, the sample types were varied, and included NPS, sputum, and BALF. The lack of standardization of the sample collecting method and site could also have affected the NGS results. To further evaluate the application of mNGS application in the diagnosis of pulmonary infections, multicenter prospective studies with a larger number of participants are required. In addition, the impact of the sample collection method and sample type on mNGS performance need further evaluation.

In conclusion, mNGS currently cannot replace conventional methods of pathogen detection, but its unbiased detection and genetic information capabilities contributed to additional diagnosis yield, making it suitable for use as a supplementary method.

## Data Availability Statement

The datasets presented in this study can be found in online repositories. The names of the repository/repositories and accession number(s) can be found at: https://db.cngb.org/cnsa/, CNP0001450.

## Ethics Statement

The studies involving human participants were reviewed and approved by Ethics Review Committee of Huashan Hospital, Fudan University. The patients/participants provided their written informed consent to participate in this study.

## Author Contributions

Y-YQ, H-YW, and YZ collected and analyzed medical data of the patients, and wrote and revised the manuscript. H-CZ, Y-MZ, XZ, and YY participated in the treatment of the patients during hospitalization and data collection. PC and H-LW participated in the next generation sequencing and data analysis. J-LJ, J-WA, and W-HZ made a critical contribution to the treatment plan of the patient and made a critical revision of the manuscript for important intellectual content. All authors contributed to the article and approved the submitted version.

## Funding

This work was supported by the National Science and Technology Major Project of China (No. 2018ZX10305409-001-001, 2018ZX10305409-001-003), National Natural Science Foundation of China (82002141), Project from Science and Technology of Shanghai (18495810600), Science and Technology Innovation Project of Shanghai (20Y11900400), Project of Shenkang (SHDC22020214) and Shanghai Youth Science and Technology Talents Sailing Project (20YF1404300).

## Conflict of Interest

H-LW was employed by BGI PathoGenesis Pharmaceutical Technology Co., Ltd.

The remaining authors declare that the research was conducted in the absence of any commercial or financial relationships that could be construed as a potential conflict of interest.
